# Prospective comparison of ^68^Ga-FAPI-04 and ^18^F-FDG PET/CT for tumor staging in nasopharyngeal carcinoma

**DOI:** 10.3389/fonc.2022.1047010

**Published:** 2022-12-08

**Authors:** Haoyuan Ding, Juan Liang, Lin Qiu, Tingting Xu, Liang Cai, Qiang Wan, Li Wang, Ya Liu, Yue Chen

**Affiliations:** ^1^ Jinan University, Guangzhou, Guangdong, China; ^2^ Department of Nuclear Medicine, The Affiliated Hospital of Southwest Medical University, Luzhou, Sichuan, China; ^3^ Nuclear Medicine and Molecular Imaging Key Laboratory of Sichuan Province, Luzhou, Sichuan, China; ^4^ Institute of Nuclear Medicine, Southwest Medical University, Luzhou, Sichuan, China; ^5^ Department of Ultrasound, The Affiliated Hospital of Southwest Medical University, Luzhou, Sichuan, China

**Keywords:** ^18^F-FDG, ^68^Ga-FAPI-04, PET/CT, nasopharyngeal carcinoma, tumor staging

## Abstract

**Purpose:**

To explore the difference in the effectiveness of gallium-68 fibroblast activation protein inhibitor (^68^Ga-FAPI-04) PET/CT and fluorine-18 fluorodeoxyglucose (^18^F-FDG) PET/CT for the initial staging of patients with nasopharyngeal carcinoma (NPC).

**Methods:**

The Affiliated Hospital of Southwest Medical University hosted this single-center prospective investigation (Clinical Trials registration No.ChiCTR2100044131) between March 2020 and September 2021. Within a week, all subjects underwent MR scans, ^68^Ga-FAPI-04 PET/CT, and ^18^F-FDG PET/CT in order. The effectiveness of medical staging employing ^68^Ga-FAPI-04 and ^18^F-FDG PET/CT was compared.

**Results:**

Twenty-eight patients with primary NPC were evaluated (mean age53 ± 11 years). ^68^Ga-FAPI-04 PET/CT indicated an elevated recognition rate for diagnosing primary tumors (28/28 [100%] vs. 27/28 [96%]) and lymph node metastases (263/285 [92%] vs. 228/285 [80%]), but a lower detection rate for distant metastases (5/7 [71%] vs. 7/7 [100%]) compared with ^18^F-FDG PET/CT. A significant association between the maximum standard uptake value (SUVmax) of ^18^F-FDG PET and ^68^Ga-FAPI-04 PET was found in the primary cancers (r = 0.691, p < 0.001). In comparison to ^18^F-FDG PET/CT, ^68^Ga-FAPI-04 PET/CT upstaged the T stage in five patients while downstaging the N stage in seven patients. ^68^Ga-FAPI-04 PET/CT corrected the overall staging of five patients on^18^F-FDG PET/CT.

**Conclusion:**

^68^Ga-FAPI-04 PET/CT is preferable to ^18^F-FDG PET/CT for NPC staging in terms of the detection efficiency for primary tumors and lymph node metastasis. This is especially true when evaluating the primary cancer and any spread to contiguous tissues. It is possible to improve the staging assessment of NPC by using ^68^Ga-FAPI-04 PET/CT in conjunction with ^18^F-FDG PET/CT.

## Introduction

Nasopharyngeal carcinoma (NPC) is a prevalent epithelial malignancy; its incidence is related to ethnicity and regional distribution. People in East and Southeast Asia, especially in Fujian and Guangdong areas of China, have a high incidence of NPC (1). In 2020, a survey of 185 countries determined that NPC was newly diagnosed in 133,354 patients and resulted in almost 80,000 fatalities (2). NPC tends to infiltrate locally early and typically involves regional nodes (3). Patients with terminal disease often develop distant metastases (4); thus, early and correct staging of NPC is critical for enhancing the individuals’ quality of life and treatment outcomes (5).

As the first-choice imaging method for NPC, MR is excellent for showing adjacent soft tissue infiltration, skull base bone and intracranial invasion, and retropharyngeal lymph node involvement (6). The National Comprehensive Cancer Network currently recommends fluorine-18 fluorodeoxyglucose (^18^F-FDG) PET/CT as a well-proven imaging strategy for NPC management (7), with elevated accuracy and sensitivity for identifying lymph nodes and distant metastases (8, 9). ^18^F-FDG reveals the glucose metabolism of abnormalities. Owing to the high physiological glucose utilization in healthy brain tissues and the lesser soft tissue resolution of PET/CT compared to that of MR, the precision of ^18^F-FDG PET/CT for the T staging of NPC is insufficient, mainly for the description of the skull base and intracranial invasion (10, 11).

Gallium-68-labeled fibroblast activation protein inhibitor (^68^Ga-FAPI) is a recently developed cancer tracer. It indicates the degree of fibroblast activation protein (FAP) expression (12–14). Cancer-associated fibroblasts overexpress FAP in most epithelial cancers, including NPC, whereas its expression is modest in most healthy tissues and organs. PET/CT using ^68^Ga-FAPI-04 reveals tumors and metastases in various malignant tumors, such as head and neck cancers, with strong tracer uptake in lesions (13–15). ^68^Ga-FAPI has a greater target to background ratio than ^18^F-FDG (16). Furthermore, prior research have demonstrated that ^68^Ga-FAPI-04 PET/CT is an effective investigative approach for NPC, especially for the assessment of the primary cancer and any spread to contiguous tissues (17, 18).

Therefore, we carried out a prospective investigation to explore the difference in the effectiveness of ^18^F-FDG PET/CT and ^68^Ga-FAPI-04 PET/CT in discovering primary tumor, nodal, and distant metastases in patients with NPC.

## Materials and methods

### Participants

Between March 2020 and September 2021, the affiliated hospital of Southwest Medical University provided access to this prospective medical trial. The research protocol was approved by both the China Clinical Trials Registry and the Clinical Research Ethics Committee at the previously mentioned hospital (Clinical Trials registration No.ChiCTR2100044131; Ethics Committee approval No.2020035). All individuals gave their written permission after being fully informed. Within seven days, all individuals completed MR scans, ^68^Ga-FAPI-04 PET/CT, and ^18^F-FDG PET/CT in order. The acquisition interval between ^68^Ga-FAPI-04 PET/CT and ^18^F-FDG PET/CT was at least one day. The criteria for inclusion were: (a) individuals with *de novo* histopathologically given a diagnosis NPC; (b) subjects participated who had not received antitumor therapy before the evaluation; (c) individuals with cancer who chose to undergo paired ^18^F-FDG along with ^68^Ga-FAPI-04 PET/CT tests to stage their disease; and (d) subjects who agreed to follow the protocol procedures, gave their written informed consent, and gave their signatures. The following is a list of the conditions for exclusion: (a) individuals with contraindications for the exams, (b) people with additional primary cancers at the time of the testing, and (c) individuals who began therapy prior to the completion of the three tests.

### ^18^F-FDG and ^68^Ga-FAPI-04 preparation

^18^F-FDG was formed utilizing normal procedures and a coincident ^18^F-FDG synthesizing form (FDG-N, PET Science & Technology). DOTA-FAPI-04 was acquired from MedChemExpress LLC. As previously mentioned (19), radiolabeling and purifying of ^68^Ga-FAPI-04 were conducted. The radiochemical purity of ^68^Ga-FAPI-04 and ^18^F-FDG exceeded 95%, and the finished radiopharmaceuticals were sterile and devoid of pyrogens.

### Imaging acquisition

Before undergoing the ^18^F-FDG PET/CT evaluation, the subjects abstained from food and drink for at least six hours to ensure that their blood glucose levels were within the accepted values (3.9–6.1 mmol/L). However, there was no need to make any preparations in order to take the ^68^Ga-FAPI-04 PET/CT test. The doses of ^68^Ga-FAPI-04 and ^18^F-FDG that were administered *via* intravenous injection were 3.7 and 1.85 MBq/kg, respectively (19, 20). Following a tracer injection, participants got a PET/CT scan (uMI780, United Imaging Healthcare) 40–60 mins later. All scans were conducted in accordance with a previously outlined technique (21, 22), and the resulting data were provided to a post-processing workstation (Version R002, uWS-MI, United Imaging Healthcare). The PET data were recreated with the help of an algorithm called sorted subset anticipation maximization (two iterations and 20 subsets). Evaluations of the nasopharynx and the cervical area employing contrast-enhanced (CE) MR were carried out using head and neck coils on 1.5-T MR scanners (Achieva 1.5T, Philips, Amsterdam, the Netherlands). We acquired the MR images, containing axial T1-weighted fast spin-echo images immediately before injection of contrast. (repetition time [TR] = 450 ms; echo time [TE] = 15 ms, flip angle = 90°, field of view [FOV] =232 mm × 232 mm, slice thickness = 5 mm, spacing between slices = 1 mm), axial T2-weighted fast spin-echo images (TR = 3,575 ms, TE = 80 ms, flip angle = 90°, FOV =232 mm × 232 mm, slice thickness = 5 mm, spacing between slices = 1 mm), and axial and coronal T2-weighted fat-suppressed spin-echo images (TR = 1,927 ms, TE = 55 ms, flip angle = 90°, FOV =250 mm × 250 mm, slice thickness = 5 mm, spacing between slices = 1 mm). At a rate of 1.5 mL/s, intravenous doses of 0.1 mmol/kg gadopentetate dimeglumine were delivered. Using the exact parameters as the axial T1-weighted fast spin-echo images, the axial T1-weighted fast spin-echo sequence was obtained.

### Imaging analysis

Two board-certified nuclear medicine specialists investigated all PET/CT sets of data. To avoid bias, cohort 1 (L.C. and Y.C.) assessed all ^18^F-FDG PET/CT pictures, while cohort 2 (Y.Z. and L.Q.) reviewed all ^68^Ga-FAPI-04 PET/CT images. Two board-certified radiologists (D.C. and J.S.) who were blinded to the PET/CT outcomes analyzed the MRI. Investigating any non-physiological uptake employing ^18^F-FDG or ^68^Ga-FAPI-04 PET that was higher than the activities of the background blood pool or the activities of the background of the neighboring healthy tissue was the primary focus of the study. On transverse PET scans, regions of interest were outlined for semi-quantitative analysis. The SUVmax was automatically computed to estimate the uptake of ^18^F-FDG or ^68^Ga-FAPI-04 in primary cancers, associated lymph nodes, and distant metastases. Clinical staging is based on three different types of images in accordance with the American Joint Committee on Cancer staging system version 8th (23).

#### Primary lesion evaluation

On PET images, the SUVmax of every primary lesion was recorded. By comparing the radioactivity of the lesion border to that of the nearby healthy tissue, the boundaries were visually evaluated. The border and extent of the invasion were identified if the radioactivity at the border of the injury was significantly greater than that of the nearby healthy tissue. Corresponding CT image was employed to help recognize morphology and localization of the lesions. The extent and border of every lesion were evaluated, and any variation between the three imaging techniques were noted.

#### Lymph node evaluation

Patients’ lymph nodes were categorized into four sections: the retropharyngeal region, the right and left sides of the neck located above the cricoid cartilage inferior edge, and the region below the cricoid cartilage inferior boundary. Employing ^18^F-FDG and ^68^Ga-FAPI-04 PET/CT, the quantity of lesions and SUVmax with the greatest pathological tracer buildup were measured for every lymph node area, and the techniques were compared. According radiographic criteria, the MR identification of metastatic lymph nodes located in the cervical region must meet at least one of the following (9): (a) there was extracapsular expansion or necrosis, (b) in the retropharyngeal region, the lowest axial diameter was 5 mm, and in other locations, it was ≥ 10 mm, and (c) there were ≥ 3 lymph nodes of borderline size.

#### Distant metastasis evaluation

Except for the primary tumor and nodal metastases, any non-physiological uptake above the activities of the background blood pool or the activities of the background of the neighboring healthy tissue on PET/CT, with or without morphological abnormalities, was classified as a possible distant metastasis. Distant metastases were also considered as positive if the signal is different from that of adjacent background tissues on MRI. Lesions with aberrant tracer uptake and MR signals were counted and localized. The SUVmax of each metastatic lesion was also recorded.

### Reference standard

Histopathological analysis of the biopsied or resected samples served as the basis for the definitive diagnosis. In accordance with the criteria of the National Comprehensive Cancer Network (7), CE-MR is the gold standard for assessing the cancer and its invasion of neighboring tissues. Due to technological and ethical constraints, histological verification of all lymph nodes and distant metastases was not achievable. Therefore, the tumor was classified as malignancy based on the confirmation of typical malignant characteristics by multimodal imaging. The duration of the follow-up was over three months. During follow-up following anti-cancer therapies, including chemotherapy, radiation, and/or targeted therapy, a considerable decrease in lesion size was determined to be malignant.

### Statistical analyses

All statistical analyses were done by employing SPSS (version 22.0; SPSS Inc.). Categorical data are represented numerically and as a percentage. The expression for continuous variables is the mean standard deviation. Using Spearman’s correlation analysis, the relationship between the kind of pathology and the degree of tracer uptake was found. Employing the paired samples t-test, the SUVmax values of the primary and metastatic lesions were compared between ^18^F-FDG and ^68^Ga-FAPI-04 PET/CT. The ^18^F-FDG SUVmax was compared between metastatic and non-metastatic lymph nodes using a t-test for independent samples. Two-tailed p-values of < 0.05 were regarded as statistically significant.

## Results

### Participant characteristics

This investigation comprised twenty-eight individuals (5 women and 23 men) aged 33–75 years (mean = 53 ± 10 years). ^68^Ga-FAPI-04 and ^18^F-FDG PET/CT were well tolerated by all subjects, and no ^68^Ga-FAPI-04-related side effects were identified. All individuals were newly diagnosed with nasopharyngeal carcinoma, in which two instances were keratinizing squamous cell carcinoma (WHO Type I), eleven patients were non-keratinizing differentiated carcinoma (WHO Type II), and fifteen patients were non-keratinizing undifferentiated carcinoma (WHO Type III). The clinical data is displayed in **Table 1**.

**Table 1:** Summary of patient basic characteristics.

Characteristics	Value
Number of patients	28
Age (year)	
Mean (average ± standard deviation)	53 ± 11
Range	33-75
Sex	
Female	5
Male	23
Histology, WHO type	
I	2
II	11
III	15

### Diagnostic effectiveness of ^68^Ga-FAPI-04 and ^18^F-FDG PET/CT for primary tumors

The PET/CT scan utilizing ^68^Ga-FAPI-04 identified all 28 primary cancers with a detection rate of one hundred percent. ^18^F-FDG PET/CT revealed 27 of the 28 primary cancers, which is a 96% detection rate. There was no indication of a greater SUVmax value for ^68^Ga-FAPI-04 PET in the primary malignancies comparing with ^18^F-FDG PET (12.1 ± 4.9 vs. 11.7 ± 4.6; *p* = 0.543) (**Table 2**). Additional comparison of the connection between the uptake of the two tracers revealed a substantial relation between the SUVmax values of ^68^Ga-FAPI-04 and ^18^F-FDG (r = 0.69, *p* < 0.001). Furthermore, there was no relation among the different histopathological kinds and the SUVmax of the two tracers (*p* > 0.05). A visual assessment of the primary lesion invasion was performed using the two tracers (**Table 3**).

**Table 2:** The SUVmax comparison between ^18^F-FDG and ^68^Ga-FAPI-04 PET/CT in primary tumor, nodal, and distant metastasis.

Index	Primary tumor	Nodal metastasis	Distant metastasis
^18^F-FDG PET/CT	11.7 ± 4.6	13.6 ± 5.5	8.3 ± 5.9
^68^Ga-FAPI PET/CT	12.1 ± 4.9	11.7 ± 5.0	6.6 ± 4.0
*p* value	0.543	0.133	0.450

**Table 3:** Visual evaluation of tumor invasion using the 3 modalities.

	Detection No.			Visual evaluation				
Lesion Invasion	MR	FDG	FAPI	FDG = FAPI	FDG > FAPI	FDG < FAPI	FDG = MR	FAPI= MR
Nasopharynx	28	27	28	27	0	0	25	25
Parapharyngeal space	18	18	18	16	0	2	15	17
Skull base bone	11	11*	11	7	1	4	7	11
intracalvarium	4	1	4	1	0	3	1	4

*Including 1 false positive case.

#### Nasopharyngeal invasion

**Figure 1:**
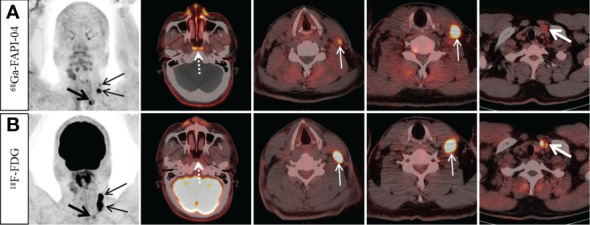
A 49-year-old man with nonkeratinizing differentiated carcinoma. ^68^Ga-FAPI-04 PET/CT showed intensive ^68^Ga-FAPI-04 uptake in the posterior nasopharyngeal wall (**A**, dotted arrow, SUVmax 3.8), ^18^F-FDG PET/CT showed no abnormal ^18^F-FDG uptake in the primary tumor (**B**, dotted arrow). Moreover, ^68^Ga-FAPI-04 PET/CT reveals higher tracer uptake than ^18^F-FDG PET/CT in the left supraclavicular lymph node (**A**, thick arrow, SUVmax 9.4 vs. **B**, thick arrow, SUVmax 3.8), but the tracer uptake of left cervical (level III) lymph nodes was lower than that of ^18^F-FDG PET/CT (**A**, thin arrow, SUVmax, 4.5–11.0 vs. **B**, thin arrow, SUVmax, 17.7–19.4).

Both modalities clearly delineated the boundary and extent of tumor invasion, except in one case (**Figure 1**) of nonkeratinizing differentiated carcinoma that was not detected by ^18^F-FDG PET. Visual evaluation of nasopharyngeal invasion was similar for the two tracers in 27 participants, but of which two cases were found to be inferior to MR.

#### Parapharyngeal space invasion

Eighteen participants had parapharyngeal space invasion. The extent of lesions on ^68^Ga-FAPI-04 PET/CT was larger than that on MR in one of the 18 participants, while there were two cases with a smaller extent and one case with a larger extent on ^18^F-FDG PET/CT compared with MR. There were 2 and 16, respectively, patients with ^68^Ga-FAPI-04 who were dominant and equal to ^18^F-FDG.

#### Skull base bone invasion

**Figure 2:**
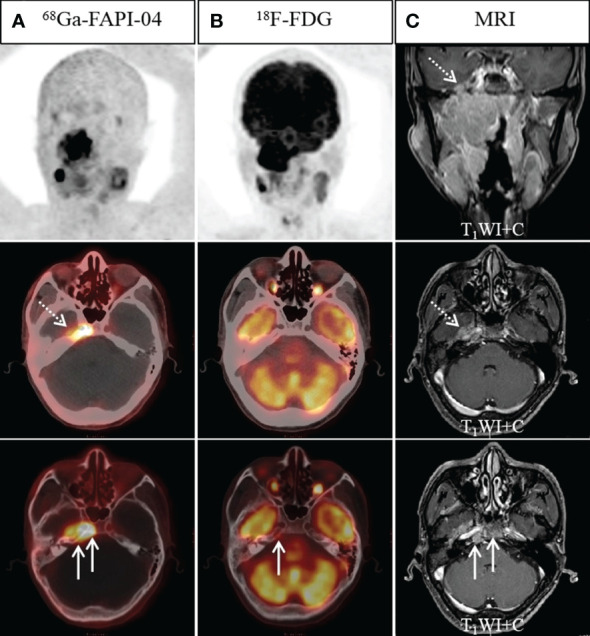
A 45-year-old man with nonkeratinizing differentiated carcinoma. Intense ^68^Ga-FAPI-04 uptake was observed in the left temporal lobe (**A**, dotted arrow), suggesting intracranial invasion, but ^18^F-FDG PET/CT **(B)** showed no abnormal intracranial ^18^F-FDG uptake. Moreover, intense ^68^Ga-FAPI-04 uptake was observed in the occipital and right temporal bone (**A**, solid arrow), while ^18^F-FDG PET/CT only showed low tracer uptake in the right temporal bone (**B**, solid arrow), which was confirmed by MRI (**C**, solid arrow). ^68^Ga-FAPI-04 PET/CT revealed more extensive lesions on intracranial and skull base invasion than ^18^F-FDG PET/CT.

Typically, 11 participants had invasion of the skull base bone. ^68^Ga-FAPI-04 PET/CT had a 100% (11/11) positive detection rate and showed a tumor extent and border delineation similar to that of MR. The 11 patients discovered by ^18^F-FDG PET/CT included one false-positive case, while one person with skull base invasion went undetected. In 4 of the 11 participants, ^68^Ga-FAPI-04 PET/CT showed a greater degree of skull base bone invasion compared to ^18^F-FDG PET/CT (**Figure 2**).

#### Intracranial invasion

Typically, 4 participants had an intracranial invasion. The positive detection rates for ^68^Ga-FAPI-04 PET/CT along with ^18^F-FDG PET/CT were, respectively, 100% (4/4) and 25% (1/4) (**Figure 2**). Owing to the physiological high uptake of ^18^F-FDG in the brain, both ^68^Ga-FAPI-04 PET/CT and MR revealed a more precise border of intracranial invasion than ^18^F-FDG PET/CT.

### Diagnostic effectiveness of ^68^Ga-FAPI-04 and ^18^F-FDG PET/CT for nodal metastasis

**Figure 3:**
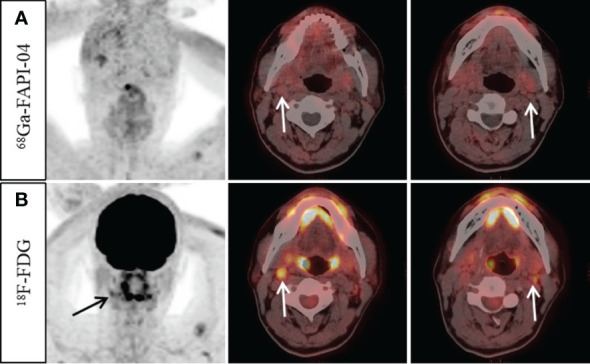
A 57-year-old man with nonkeratinizing undifferentiated carcinoma. An increase ^18^F-FDG uptake was observed in the bilateral cervical (level II) lymph nodes (**B**, arrow, SUVmax 3.3–4.2). However, no abnormal ^68^Ga-FAPI-04 uptake was observed in the cervical lymph nodes (**A**, arrow). Ultrasound-guided biopsy of the right level II lymph node revealed proliferating lymphoid cells with no signs of metastatic disease. Finally, it was confirmed by follow-up that all the suspected metastatic lymph nodes were reactive.

Twenty-seven of the 28 participants (285 lymph nodes) were suspected to have lymph node metastases. For 25/285 lymph nodes, histopathological analysis acted as a reference standard, and for the remaining lymph nodes, morphological analysis and/or follow-up imaging were used. Of the 285 suspected lymph nodes, 234 lymph nodes in 24 participants were considered malignant and 51 lymph nodes were lastly verified as inflammatory. From a total of 285 lymph nodes, ^68^Ga-FAPI-04 PET/CT recognized 263 (false-positive uptake in 2 lymph nodes and false-negative uptake in 20 lymph nodes). By comparison, 228 lymph nodes were successfully detected by ^18^F-FDG PET/CT (false-positive uptake in 51 lymph nodes and false-negative uptake in six lymph nodes, **Figure 3**). MR accurately diagnosed 262 lymph nodes, with two false-positive and twenty-one false-negative lymph nodes, respectively. Only one of the 203 lymph nodes with positive ^68^Ga-FAPI-04 and ^18^F-FDG uptake was confirmed to be a false positive. The SUVmax of metastatic lymph nodes was somewhat greater in ^18^F-FDG than in ^68^Ga-FAPI-04 (13.6 ± 5.5 vs. 11.7 ± 5.0), but the variation was not substantially significant (*p* = 0.133. **Table 2**). Significantly greater ^18^F-FDG uptake was seen in the metastatic lymph nodes compared to the non-metastatic reactive lymph nodes (13.6 ± 5.5 vs. 3.2 ± 0.7; *p* < 0.001).

### Diagnostic effectiveness of ^68^Ga-FAPI-04 and ^18^F-FDG PET/CT for distant metastasis

**Figure 4:**
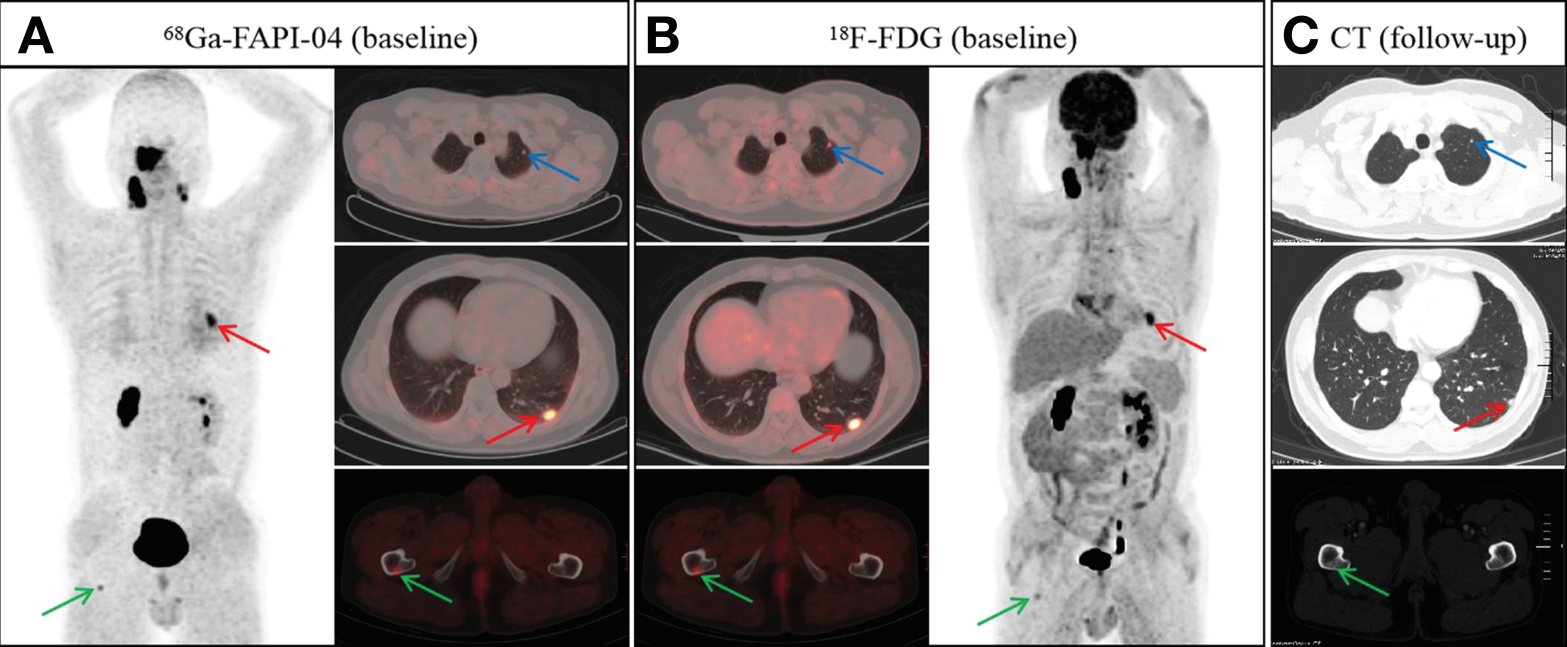
A 48-year-old man with nonkeratinizing undifferentiated carcinoma.^68^Ga-FAPI-04 **(A)** and ^18^F-FDG PET/CT **(B)** revealed an abnormal nodule in the left lower lobe (**A**, red arrow, SUVmax 4.9 vs. B, red arrow, SUVmax 8.2). However, the nodule in the left upper lobe showed abnormal uptake on ^18^F-FDG PET/CT (**B**, blue arrow), but not on ^68^Ga-FAPI-04 PET/CT (**A**, blue arrow). In addition, both ^68^Ga-FAPI-04 (**A**, green arrow) and ^18^F-FDG PET/CT (**B**, green arrow) revealed abnormal activity in the right femur. Follow-up CT after two cycles of induction chemotherapy showed a reduction in the volume of pulmonary metastases (**C**, red and blue arrow). Meanwhile, the bone metastasis of the right femur revealed a repair response after treatment, showing osteosclerotic nodule on follow-up CT (**C**, green arrow).

Among the 28 participants, seven distant metastases were found in four participants (including three pulmonary and four bone metastases). All distant metastases were detected by ^18^F-FDG PET/CT, whereas ^68^Ga-FAPI-04 uptake was negative in two pulmonary metastases (**Figure 4**). One individual had concurrent bone and lung metastases. Across all evaluations of distant metastases, the SUVmax of ^68^Ga-FAPI-04 did not vary significantly from that of ^18^F-FDG (6.6 ± 4.0 vs. 8.3± 5.9; *p* = 0.450. **Table 2**).

### Variations in tumor staging

The outcomes of the ^68^Ga-FAPI-04 PET/CT, ^18^F-FDG PET/CT, and MR imaging were compiled in **Table 4**, which provides a summary of the cancer staging for each of the 28 subjects. ^68^Ga-FAPI-04 PET/CT underestimated the number of participants in the N staging and M staging by 1. In contrast, ^18^F-FDG PET/CT undervalued the T staging in five participants and overestimated the N staging in seven participants. For the overall staging, although ^68^Ga-FAPI-04 PET/CT underestimated the medical stage of two participants, it correctly upgraded the medical staging of ^18^F-FDG PET/CT in two participants (from III to IVA) and downgraded the medical staging of ^18^F-FDG PET/CT in three participants (two from III to I and one from III to II).

**Table 4:** Comparison of MR, ^18^F-FDG, and ^68^Ga-FAPI-04 PET/CT-based tumor staging (n = 28).

ParticipantNo.	Tumor Stage(FDG-based)	Tumor Stage(FAPI-based)	Tumor Stage(MR-based)	Additional finding(FDG vs FAPI)	Additional finding(FAPI vs FDG)	Staging changes (FAPI vs FDG)
1	III: T1N2M0	I: T1N0M0	I: T1N0M0	None	None	Down
2	III:T3N2M0	IVA: T4N2M0	IVA: T4N2M0	Cervical lymph node	Intracranial involvement	Up
3	IVB: T2N3M1	IVB: T2N3M1	IVA: T2N3M0	Cervical lymph node	None	None
4	IVB: T3N3M1	IVB: T2N2M1	III: T2N2M0	1 pulmonary metastasis	None	None
5	III: T2N2M0	II: T2N1M0	III: T2N2M0	Cervical lymph node	None	Down
6	III: T3N2M0	III: T3N2M0	III: T3N2M0	None	None	None
7	III: T2N2M0	III: T3N1M0	III: T3N1M0	Cervical lymph node	Skull base bone involvement	None
8	III: T3N2M0	III: T3N2M0	III: T3N2M0	None	None	None
9	III: T2N2M0	III: T2N2M0	III: T2N2M0	None	Cervical lymph node	None
10	III: T1N2M0	III: T1N2M0	II: T1N1M0	None	None	None
11	III: T3N1M0	IVA: T4N0M0	IVA: T4N0M0	None	Intracranial involvement	Up
12	III: T3N1M0	III: T3N1M0	III: T3N1M0	None	None	None
13	III: T2N2M0	III: T2N2M0	III: T2N2M0	None	None	None
14	IVA: T1N3M0	IVA: T1N3M0	IVA: T1N3M0	None	None	None
15	II:T1N1M0	II:T1N1M0	II:T1N1M0	None	None	None
16	III: T1N2M0	I: T1N0M0	I: T1N0M0	None	None	Down
17	II:T2N0M0	II:T2N0M0	II:T2N0M0	None	None	None
18	IVA: T4N3M0	IVA: T4N3M0	IVA: T4N3M0	None	Cervical lymph nodes	None
19	III:T1N2M0	III:T1N2M0	III:T1N2M0	None	None	None
20	IVB:T3N2M1	III: T3N2M0	III: T3N2M0	1 pulmonary metastasis	None	Down
21	IVB:T3N3M1	IVB:T4N3M1	IVA: T4N2M0	None	Intracranial involvement	None
22	III:T1N2M0	III:T1N2M0	III:T1N2M0	Cervical lymph node	None	None
23	III:T1N2M0	III:T1N2M0	III:T1N2M0	Cervical lymph node	None	None
24	III: T1N2M0	II:T1N1M0	II:T1N1M0	Cervical lymph node	None	Down
25	II:T2N1M0	II:T2N1M0	II:T2N1M0	None	None	None
26	IVA: T3N3M0	IVA: T3N3M0	IVA: T3N3M0	None	None	None
27	III:T3N2M0	III:T3N1M0	III:T3N1M0	Cervical lymph node	None	None
28	IVA:T0N3M0	IVA: T1N3M0	IVA: T1N3M0	None	Primary lesion	None

## Discussion

Accurate staging is vital for NPC management. In our investigation, ^68^Ga-FAPI-04 PET/CT revealed more recognition efficiency in diagnosing primary cancers (28/28 [100%] vs. 27/28 [96%]) and lymph node metastases (263/285 [92%] vs. 228/285 [80%]) than^18^F-FDG PET/CT. However, in comparison to the efficacy of ^18^F-FDG PET/CT in identifying distant metastases, the effectiveness of ^68^Ga-FAPI-04 PET/CT in this regard was lower (5/7, or 71%), coming in at (7/7, or 100%). The combination of ^18^F-FDG along with ^68^Ga-FAPI-04 PET/CT led to consistent staging in 21 of the 28 participants, with a concordance rate of 75% for overall staging. Despite the fact that ^68^Ga-FAPI-04 PET/CT underestimated the clinical staging in 2/28 subjects, it corrected the staging of ^18^F-FDG PET/CT in 5/28 participants. As a consequence of this, we believe that ^68^Ga-FAPI-04 PET/CT is valuable for NPC diagnosis and staging.

Between the primary tumors’ ^18^F-FDG as well as ^68^Ga-FAPI-04 uptake, there was no substantial variations. Interestingly, although the imaging principles of the two tracers were different, our study revealed a significant relation between the primary tumor uptake of ^68^Ga-FAPI-04 and ^18^F-FDG, which differed from previous reports (17, 18). Raised ^68^Ga-FAPI-04 uptake in tumors is accompanied by higher glucose metabolism, which is positively correlated with cancer aggressiveness (24, 25). This demonstrates that NPC invasiveness may be predicted employing ^68^Ga-FAPI-04 imaging. Previous studies have shown that ^68^Ga-FAPI-04 PET/CT may increase the recognition rate of primary tumors in FDG-negative head and neck tumor (17, 26). In our investigation, ^18^F-FDG could not detect the primary lesion in one patient with squamous cell carcinoma confirmed by biopsy, whereas ^68^Ga-FAPI-04 successfully recognized the primary site in the posterior nasopharyngeal wall. This may be due to the superior tumor-to-background ratio of ^68^Ga-FAPI-04 PET/CT comparing with ^18^F-FDG, which may enhance the recognition rate of occult NPC.

A high physiological uptake of ^18^F-FDG in normal brain tissue may lead to an underestimation of the presence or extent of tumor invasion on PET/CT (3, 17, 18). Due to the extra benefit of a low brain background, our results confirmed that ^68^Ga-FAPI-04 dominates ^18^F-FDG PET/CT in determining malignancy invasion of the parapharyngeal space, skull base bone, and intracranial areas. At present MR is the standard approach for T staging in NPC (7). However, two participants in our study showed a smaller extent of nasopharyngeal invasion on ^68^Ga-FAPI-04 PET/CT than on MR, which may be edema and inflammation rather than tumor invasion, resulting in a positive result on MR (27). Therefore, ^68^Ga-FAPI-04 PET/CT indicated a greater recognition efficiency than ^18^F-FDG PET/CT for precise T staging, and it was able to detect the target extent for radiotherapy with a greater degree of precision (17, 18).

Staging of the nodes is essential for the management and prognostication of NPC cases. Because of the high prevalence of inflammatory and reactive hyperplasia in cervical lymph nodes, ^18^F-FDG PET/CT has been mentioned to have a greater incidence of producing false-positive outcomes when diagnosing lymph node metastasis (28, 29). In our investigation, the quantity of false-positive lymph nodes on ^18^F-FDG PET/CT was substantially more than that on ^68^Ga-FAPI-04 PET/CT and MR (^18^F-FDG, 51/279 vs. ^68^Ga-FAPI, 2/216 vs. MR, 2/215), which is consistent with the outcomes of prior investigations (28, 29). Utilising ^68^Ga-FAPI-04 PET/CT, the N staging of the 7 participants was downstaged in comparison to ^18^F-FDG PET/CT. However, ^68^Ga-FAPI-04 PET/CT revealed more metastatic lymph nodes without positive tracer uptake than ^18^F-FDG PET/CT (20/285 [7%] vs. 6/285 [2%]). Moreover, only one was diagnosed as a false positive out of all 203 lymph nodes with double-positive ^18^F-FDG and ^68^Ga-FAPI-04 uptake. Previous research has discovered significant correlations between the uptake of ^18^F-FDG and ^68^Ga-FAPI-04 in pairs of double-positive lymph nodes (18). Therefore, in assessing lymph node status in NPC patients prior to treatment, it’s possible that the specificity of ^68^Ga-FAPI-04 PET/CT is greater than that of ^18^F-FDG PET/CT. When used in conjunction with one another, ^68^Ga-FAPI-04 and ^18^F-FDG PET/CT imaging have the potential to increase diagnostic precision for lymph node metastasis in NPC cases.

NPC is prone to distant metastasis (4). Prior investigations have shown that ^68^Ga-FAPI-04 PET/CT has greater sensitivity than ^18^F-FDG in identifying visceral and bone metastases of various malignant tumors, including NPC, and metastatic lesions showed higher tracer uptake on ^68^Ga-FAPI-04 PET/CT (15, 17, 18, 21). However, in our investigation, ^68^Ga-FAPI-04 PET/CT did not have a greater detection efficiency compared to ^18^F-FDG for distant metastatic lesions in NPC. There were no substantial variations in the uptake of ^68^Ga-FAPI and ^18^F-FDG by metastatic lesions. This is likely due to the small amount of metastatic lesions that were studied (n = 7). In addition to this, the ^68^Ga-FAPI-04 PET missed both of the patient’s pulmonary metastases. Therefore, it is essential to consider false-negative status in pulmonary metastases for M staging when conducting ^68^Ga-FAPI-04 PET/CT.

Our investigation has some limitations. First, the sample size (n = 28) was small, and the number of distant metastatic lesions was particularly low. Consequently, prospective studies with greater cohorts are necessary, especially for the detection of distant metastasis. The morphologic and/or follow-up imaging data also served as the evaluation criterion in our examination because histological verification was not probable for totally nodal and distant metastases owing to ethical and technical considerations. Potential false-negative lesions were not sufficiently assessed, as imaging evaluation was also employed as a reference for cancer staging,

In summary, our preliminary findings suggest that ^68^Ga-FAPI-04 has a positive impact on the clinical stage of NPC. Since ^68^Ga-FAPI-04 PET/CT had better tumor-to-background contrast than ^18^F-FDG and less false-positive uptake in inflammatory and reactive proliferative lymph nodes, it improved the capability to recognize primary cancer and lymph node metastases, mainly for the assessment of the skull base and intracranial invasion. Nevertheless, when it comes to the detection of distant metastases, ^68^Ga-FAPI-04 PET/CT does not have an advantage over ^18^F-FDG PET/CT. The staging assessment of NPC may be improved utilizing ^68^Ga-FAPI-04 PET/CT in conjunction with ^18^F-FDG PET/CT.

## Data availability statement

The original contributions presented in the study are included in the article/supplementary material. Further inquiries can be directed to the corresponding authors.

## Ethics statement

The studies involving human participants were reviewed and approved by The Clinical Research Ethics Committee of the Affiliated Hospital of Southwest Medical University. The patients/participants provided their written informed consent to participate in this study.

## Author contributions

HD and JL contributed to the study conception and design. Material preparation and data collection were performed by HD, JL, LQ, TX, LC, QW, LW, YC. HD and YL processed and analysed the data. The first draft of the manuscript was written by HD and JL reviewed and revised the manuscript. All authors contributed to the article and approved the submitted version
